# Unforeseen current and future benefits of uncommon yeast: the *Metschnikowia* genus

**DOI:** 10.1007/s00253-024-13369-y

**Published:** 2024-12-11

**Authors:** Ariranur Haniffadli, Yeongjun Ban, Endang Rahmat, Chang Ho Kang, Youngmin Kang

**Affiliations:** 1 Korean Medicine Convergence Science Major of KIOM School, University of Science and Technology (UST), Daejeon, 34113 Republic of Korea; 2 Herbal Medicine Resources Research Center, Korea Institute of Oriental Medicine (KIOM), 111 Geonjae-Ro, Naju-Si, Jeollanam-Do 58245 Republic of Korea; 3 Biotechnology Department, Faculty of Engineering, Bina Nusantara University, Jakarta, 11480 Indonesia; 4 Plant Molecular Biology and Biotechnology Research Center, Gyeongsang National University, Jinju, Gyeongnam 52828 Republic of Korea

**Keywords:** *Metschnikowia*, Yeast, Utilization in human, Secondary metabolite

## Abstract

**Abstract:**

*Metschnikowia*, the single-cell yeast form, is a genus of 85 species in the Saccharomycetales order that developed in both aquatic and terrestrial ecosystems after being found in 1899. This yeast is commonly used to control microbial populations in many biological and artificial conditions, such as fermentation. However, current study of *Metschnikowia* is limited to biological control features rather than researching on lucrative sectors such as beverage production, bioconversion manufacturing, cosmetics, and the pharmaceutical industry. This review summarizes numerous possible applications of *Metschnikowia* in human life, including potential secondary metabolites in industrial fields such as cosmetics and pharmaceuticals. Furthermore, *Metschnikowia*-yeast interaction is mentioned as a potential area for further exploration in terms of co-cultured microbes as biocontrol. Since *Metschnikowia* yeast arose in a variety of ecosystems, more discussion will be held regarding the interactions between *Metschnikowia* and their surroundings, particularly in fruits. Finally, the current regulatory challenges of *Metschnikowia*-based products are examined, and future research opportunities on *Metschnikowia* utilization are presented.

**Key points:**

• *Utilization of Metschnikowia genus in various human aspects*.

• *Promising secondary metabolites produced by Metschnikowia*.

• *Challenge and opportunity on developing Metschnikowia-based products*.

**Graphical Abstract:**

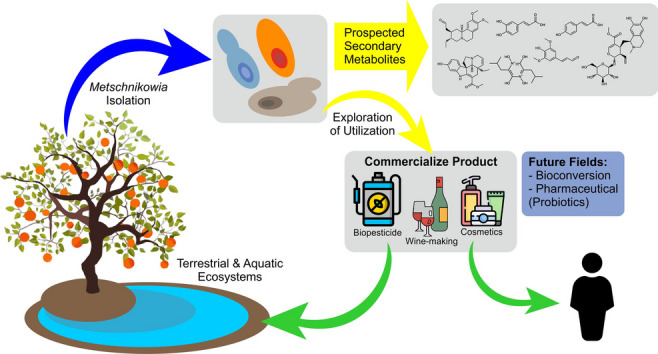

**Supplementary Information:**

The online version contains supplementary material available at 10.1007/s00253-024-13369-y.

## Introduction

Yeasts are a natural resource used in various fields of human life. Since ancient times, yeast has been a necessary component for both fermentation and flavor enhancement in fermented foods such as cheese, yogurt, and bread (Tofalo et al. 2020). Moreover, yeast has significantly contributed to pharmaceutical production, biomanufacturing, and environmental control (Ali et al. 2024; Maiguma et al. 2024; Tix et al. 2024). For instance, *Saccharomyces cerevisiae*, a traditional yeast, has been widely used as a biosensor for monitoring tebuconazole in the environment, producing biopharmaceuticals such as insulin, glucagon, and human papiloma virus (HPV), and synthesizing therapeutic natural compounds such as scopolamine, taxadiene, and resveratrol (Kulagina et al. 2021; Mendes et al. 2024). In addition, researchers have utilized *Pichia pastoris* for bioconversion in the production of non-food ingredients (Guo et al. 2022; Meng et al. 2023). Several *Metschnikowia* species are non-traditional yeast strains that have been underestimated for their value, despite being effectively utilized in cosmetic manufacturing, enology, and crop biological control (Paufique 2019; Roudil et al. 2020; Vicente et al. 2020).

**Fig. 1:**
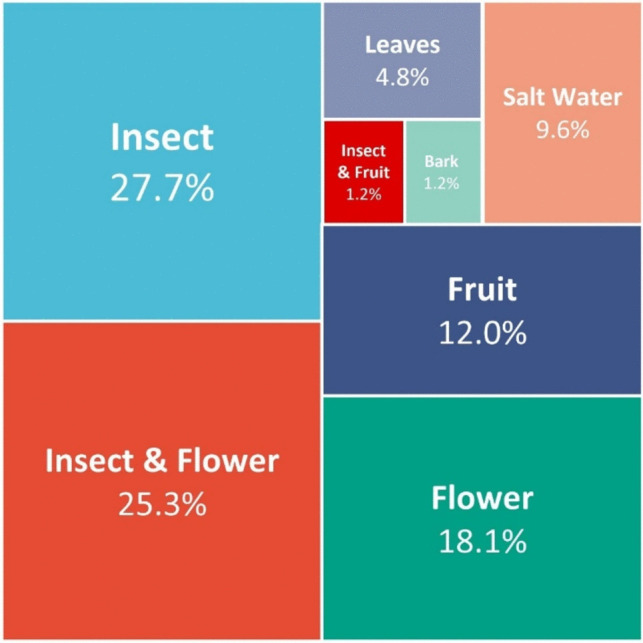
Ecological distribution of initial discovery of *Metschnikowia* species

The discovery of new *Metschnikowia* strains has surged over the past century. The genus was initially identified approximately 125 years ago, and now encompasses 85 species, with advancements in the identification and characterization of microorganisms (Table S1**.**) (Mycobank and indexfungorum database, 16 Nov 2023). Of the 85 species, 23 were isolated from insects (27.7%), 15 from plant flowers (18.1%), and 21 from insect-flower ecosystems, with others discovered in various plant parts, including fruits, bark, leaves, and aquatic environments through modern technological approaches (Fig. 1) (Kurtzman et al. 2018). The utilization of *Metschnikowia* yeast in human life has expanded over the past two decades. Advanced technologies have revealed several biological features of *Metschnikowia*, highlighting their importance in the agricultural, enology, and cosmetic industries.

Therefore, to maximize the efficiency of *Metschnikowia* species utilization, a deeper understanding of their characteristics and current utilization status is essential. This review aimed to collect information on commercial products utilizing *Metschnikowia* yeast, detail their current uses, and emphasize their potential use across various fields and ecological roles. In addition, this review provides fundamental knowledge for research groups that investigate *Metschnikowia* yeast, especially *Metschnikowia persimmonesis* which was discovered in 2017 in our laboratory (Kang et al. 2017). This review is divided into several sections. The first describes the current functional classification of *Metschnikowia* based on previous research. The next section explores the prospected secondary metabolites produced by this yeast species. Thereafter, this review present interaction between *Metschnikowia* species with other yeast as potential topic to investigate, particularly biological control sectors, as well as a section on the role of *Metschnikowia* in fruit ecosystem, because almost all *Metschnikowia*-related products target fruit-growing ecosystems. Moreover, the interactions of region-specific *Metschnikowia* strains with their environments, discussing the benefits and prospects for their utilization. This includes insights from research on *M. persimmonesis* and *M. koreensis* isolated from ecologically unique areas in South Korea. The last chapter explores the present research trends and suggests potential paths for the ongoing study of this fascinating yeast species in a variety of contexts.

## Functional classification of *Metschnikowia*

**Fig. 2:**
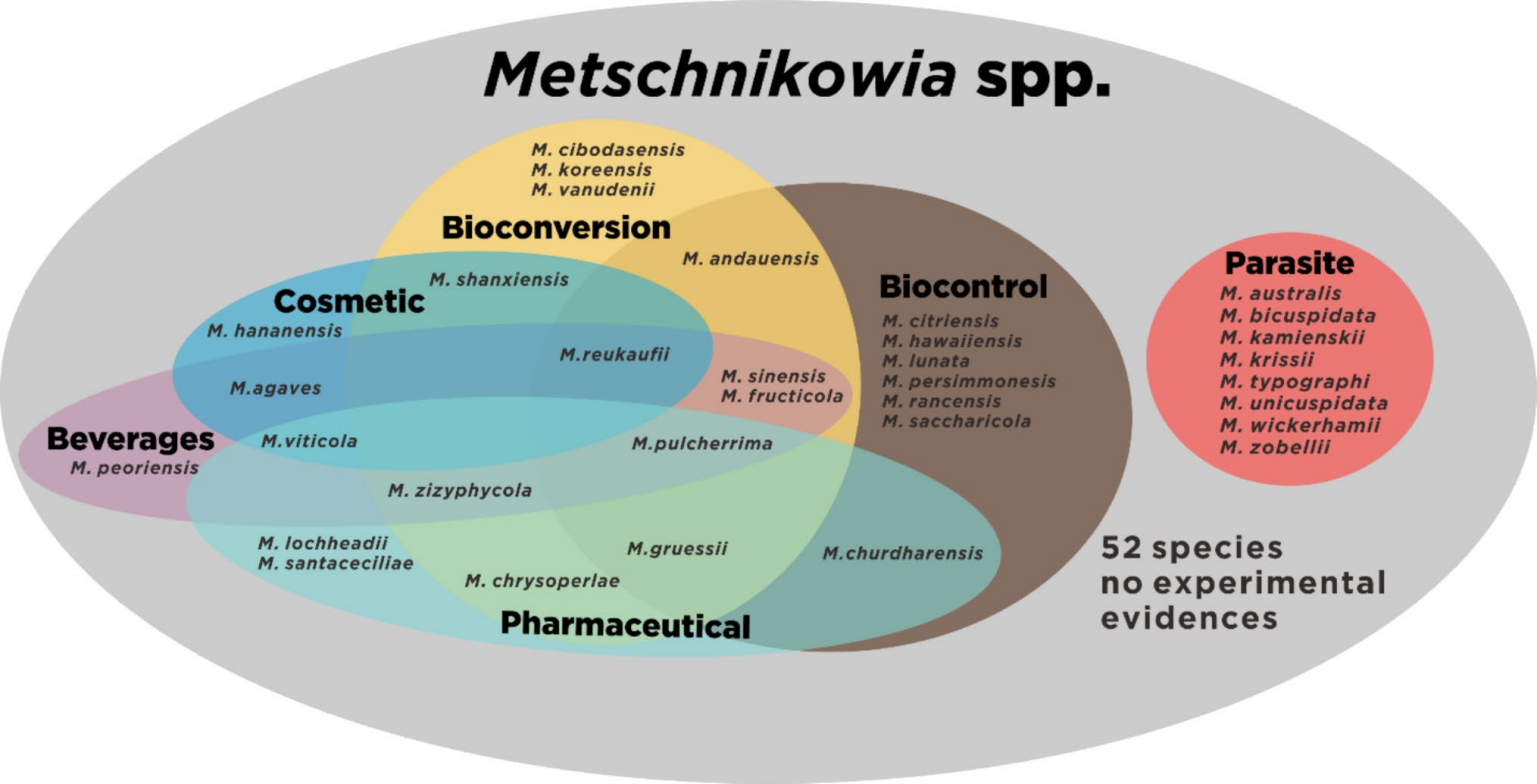
Classification of *Metschnikowia* species based on utilization in human life

Since the discovery of *Metschnikowia* species, various functional properties of each species have been tested. The functional characteristics of *Metschnikowia* can be used as additional features to distinguish between individual species (Fig. 2). The classification of *Metschnikowia* species based on their functional traits could be valuable in determining how the genus *Metschnikowia* could be utilized by humans in the future. Categorization of the functional *Metschnikowia* could bridge the gap in the application of this genus in different areas of research. In addition, this grouping may provide a better understanding of the reasons for the slow progress in certain downstream research areas.

### Parasitism

The initial exploration of the genus *Metschnikowia* focused on its parasitic interactions with host organisms in ecological niches (Kamienski 1899). Subsequent discoveries of *Metschnikowia* species involved in parasitic interactions further strengthened the assumption that *Metschnikowia* is a parasitic yeast genus (Codreanu and Codreanu-Balcescu 1981). Recent studies, however, have revealed beneficial aspects of *Metschnikowia* species. Technological advancements have improved our understanding of the beneficial interactions between *Metschnikowia* yeast species and their host organisms. For instance, *Metschnikowia saccharicola* shows antimicrobial activity against *Metschnikowia bicuspidata*, the pathogenic yeast isolated from salt-water, which causes milky sickness in crabs (Fig. 2) (Tan et al. 2018b). This species produces a killer toxin protein kinase that selectively disrupts cell wall integrity and leads to death (Tan et al. 2018a). This investigation demonstrates that *Metschnikowia* isolated from aquatic environments do not always exclusively exhibit negative interactions. A thorough investigation of the parasite genetics of *Metschnikowia* may provide insights into its pathogenic mechanisms, potentially inspiring researchers to develop planned deployment strategies against infectious yeasts in aquaculture systems. According to our literature study, no scientific evidence indicates the use of *Metschnikowia* as model parasites in aquatic environments. Therefore, transcriptomic and genomic analyses could position *Metschnikowia* as a model yeast pathogen in aquatic ecosystem to further enhance our understanding of its interactions with host species and the characteristics of the disease caused by it, which the same approach had been used on various organisms (Chen et al. 2023; Ovchinnikova and Shi 2023; Pierlé et al. 2012; Yu et al. 2022). Furthermore, advanced chromatography techniques, such as ultra-performance liquid chromatography (UPLC) tandem with various detectors, could enhance our understanding of killer toxin arrangement before molecular biology strategies are employed to increase toxin production (Doekes et al. 2019; Goessens et al. 2021). Tan et al. (2018a) effectively defined the amino acid sequences of pure killer toxin produced by *M. saccharicola* DD21-2, analogous with protein kinase from *S. cerevisiae* S288c, and subsequently optimized production based on this discovery (Tan et al. 2018b). Additionally, toxicity assessments are essential before considering the future application of killer toxins in aquatic environments, since the potential detrimental effect of various toxins types are emphasized by Thi et al. (2024) in their review.

### Biological control

The microbial biological control activity of *Metschnikowia* species is its most extensively studied property and forms the foundation for further exploration in various fields (Fig. 2) (Oztekin and Karbancioglu-Guler 2021; Rahmat et al. 2024). Initial experiments demonstrated the capability of *Metschnikowia* to suppress the growth of unfavorable organisms in horticultural plants during postharvest processing (Steglińska et al. 2022; Zhang et al. 2023). Utilization of *Metschnikowia* as a bioprotectant has resulted in a commercial product (Table 1.), which is applied before the harvesting period to mitigate disease severity in the final products during storage and shipping. Biopesticide evaluation is critical in agriculture, and this new trend is expected to explore non-conventional yeasts as novel biocontrol organism candidates (Steglińska et al. 2022). *Metschnikowia* species exhibit various mechanisms to combat plant pathogens, such as resistance induction, nutritional and growth condition modulation, direct antagonist action, and metabolic cascade regulation to produce bioactive compounds, which demonstrated by *Metschnikowia pulcherrima* during invitro examination against apple, grape, peach, and potato pathogens (Bühlmann et al. 2021; Curtis et al. 1996; Millan et al. 2022; Steglińska et al. 2022; Wang et al. 2020). Despite the commercialization of *Metschnikowia* products, research gaps exist regarding their biocontrol effect, particularly on the dynamic development of environmental microbial communities. Ferraz et al. (2019) implied that utilization of yeast-based biocontrol agents is safe since the organisms are naturally occurring and the secreted substances will not persist long enough to be toxic to other living organisms. However, long-term application of biological control across varied environments may influence other beneficial microbes and disturbance of the microbial ecological equilibrium, which requires additional exploration in a diverse environment condition to support mathematical model of this issue conducted by Reilly and Elderd (2014). Furthermore, Cavalcanti et al. (2020) a favorable interaction between biocontrol agents and garlic in the synthesis of active compounds, which should be investigated further in other plant.

**Table 1:** List of commercial products of *Metschnikowia*

Species	Industrial field	Activity	Commercial brand name	Ref
*Metschnikowia agaves*	Cosmetic	Anti-wrinkle	PROHYAL + ®	Paufique (2013)
*Metschnikowia fructicola*	Enology	Bioprotectant	Gaïa™	Roudil et al. (2020)
	Agriculture	Prevent severity of plant disease	Noli®, Shemer ®	Kurtzman and Droby (2001)
*Metschnikowia pulcherrima*	Enology	Bioprotectant	Excellence® B-NATURE®, LEVEL2 Initia™ PRIMAFLORA® VB BIO ZYMAFLORE® KHIO^MP^	Puyo et al. (2023b), Roudil et al. (2020)
		Enhance aroma compound	LEVEL2 Flavia™,	Vicente et al. (2020)
*Metschnikowia reukaufii*	Cosmetic	Rebalance skin microbiota	ECOBIOTYS®	Paufique (2019)

### Beverage production

*Metschnikowia*, one of the non-traditional *Saccharomyces* wine yeast genera, plays a significant role in wine making, and has been studied extensively (Fig. 2) (Boscaino et al. 2019; Morera et al. 2022; Puyo et al. 2023c; Staniszewski and Kordowska-Wiater 2023). *Metschnikowia*’s application extends beyond alcohol production to enhance flavor and aroma, which are critical considerations in winemaking, as well as to prevent undesired microbial growth during fermentation (Binati et al. 2020, 2023; Boscaino et al. 2019; Canonico et al. 2023; Puyo et al. 2023c; Varela et al. 2021). *M. pulcherrima* demonstrates these two capabilities; it has been commercialized as an efficient additional component in the fermentation process for limiting microbial spoilage while simultaneously improving the scent of the wine product (Table 1) (Canonico et al. 2023; Puyo et al. 2023c). *M. pulcherrima*, extensive species studied in winemaking, is selected for its non-antagonistic behavior toward *Saccharomyces cerevisiae* during fermentation (Vicente et al. 2020). In a comprehensive review of the *Metschnikowia* genus in wine making, Vicente et al. (2020) identified eight fermentation parameters: fermentation capacity; glycerol content; nitrogen metabolism; total acids; volatile acidity; aroma compound; polysaccharide, and mannoprotein content; color, anthocyanin, and polyphenol content. However, the available commercial products on the market emphasize the strategic application of *Metschnikowia* as a bioprotectant over other attributes (Table 1). In contrast, various research investigation on combination of *Metschnikowia* (Boscaino et al. 2019; Liu et al. 2017) with *Saccharomyces* in wine making lies in enhancing wine aroma as the main objective rather than biological control during fermentation, which is probably related to generate hydrolytic enzymes ability (Jolly et al. 2014). Marketing strategy probably a background for the highlighting the bioprotectant function as a result of the regulation prohibiting the use of fungicides prior to the harvest process of the fruit for wine making (Vicente et al. 2020). Therefore, other *Metschnikowia* species relevant to wine production should be investigated further as bioprotectants, olfactory enhancers, or other features, as long as they do not compete with *Saccharomyces* yeast.

### Cosmetics manufacturing

Notably, the antibacterial activity of *Metschnikowia* serves not only as a biocontrol in agriculture, but also as an active ingredient in cosmetics. The cosmetics industry uses *Metschnikowia agaves* and *Metschnikowia reukaufii* for their diverse activities related to the skin barrier function (Table 1**, **Fig. 2**)** (Paufique 2013, 2019). *Metschnikowia reukaufii* extracts contain peptides, sugars, and minerals that act as active ingredients in healing skin disorders by resetting the skin microbiota (Paufique 2019). Additionally, *M. agaves*’ hydrolase, identified as an active component, contains oligosaccharides with degrees of polymerization ranging from 2 to 17 (Paufique 2013). These components naturally increase the production of hyaluronic acid and hyaluronic acid synthase 2 (HAS2), resulting in wrinkle reduction and skin surface conformation (Paufique 2013). These findings highlight the potential of non-pathogenic microbes as novel ingredients in skincare. Commercial products derived from other *Metschnikowia* species from terrestrial environments may reveal additional skin barrier-related attributes. Various *Metschnikowia*-based terrestrial ecosystems (Fig. 1), such as fruits and flowers, might produce distinct secondary metabolites beneficial for the dermal surfaces of the skin. For instance, *M. reukaufii* isolated from flowers produces a poisonous chemical to control other microbiomes, and then dominate nutrients in the ecosystem; therefore, it might play a role in balancing skin microbiota activity (Mim et al. 2024; Pozo et al. 2012). It is anticipated that the use of yeast-based materials in cosmetics will continue to increase, as increasingly stringent selection criteria are considered to ensure safe ingredients and high-quality products (Khan and Abourashed 2011). In addition, Gupta et al. (2019) supported that implementing of natural resources as cosmetic ingredients could reduce any potential adverse effects of the products. Furthermore, as there are no hazardous components or high safety technology requirements, the prospective *Metschnikowia* species’ non-pathogenic attributes might become a primary necessity in the skincare sector especially as skin microbiome-friendly ingredients (Mim et al. 2024; van Belkum et al. 2023).

### Bioconversion

Several *Metschnikowia* species are known for their ability to produce compounds linked to oleaginous substances (Fig. 2) (Kumari et al. 2021; Nemcova et al. 2021; Sitepu et al. 2014). According to Nemcova et al. (2021), oleaginous and oleogenic yeasts accumulate lipids under diverse conditions. *Metschnikowia* genus is known to transform raw materials into valuable substances for humans, such as intermediates and commercial products. Since the majority of this genus consists of non-pathogenic yeast, they offer significant advantages for biotransformation and bioproduction of beneficial lipids used in food, fuel, and various industrial applications. For instance, seven *Metschnikowia* species generate lipids from waste substrates, such as animal fat, resulting in diverse fatty acid contents (Nemcova et al. 2021). Furthermore, in a stable semi-continuous pilot-scale system, *M. pulcherrima* contributed to the transformation of starch hydrolysate into a lipid molecule containing 27.6% (w/w) fatty acids (Abeln et al. 2020). In addition, Abomohra et al. (2021) showed an excellent biodiesel characteristic which is converted from the hydrolysate of food waste supplemented with Rice straw and softwood sawdust using *M. pulcherrima.* These findings present novel approaches for lipid production relevant to energy and manufacturing sectors. Furthermore, *Metschnikowia* contributes to the bioconversion of alcohol chains in various substances, which is beneficial across various industries. Examples include the synthesis of L-arbitol from L-glycerol, L-psicose from L-talitol, and the stereo inversion of alcohol diols (Meena et al. 2014; Nozaki et al. 2003; Sasahara and Izumori 2005). However, further investigations are required to address metabolic burden during transformation reactions which is a typical challenge for selected yeast (e.g., *S. cerevisiae* and *P. pastoris*) as cell factories (Cai et al. 2022; Duperray et al. 2024; Mao et al. 2024). Environmental optimization is crucial in bioconversion processes, encompassing factors like substrate compatibility (highlighted by Abomohra et al. (2021)) and microbiological contamination from the laboratory to the production scales. Brexó and Sant’Ana (2017) emphasized the unfavorable effects of contamination, including cell flocculation and a loss in both cell viability and process yield. *Metschnikowia*’s biocontrol properties could minimize microbial contamination during lipid bioproduction, leading it to be a fascinating prospect for bioconversion processes (Sitepu et al. 2014).

### Pharmaceutical applications

The pursuit of novel pharmacological entities derived from yeast encompasses two distinct approaches: the first involves the traditional isolation of a novel pharmaceutical substance, while the second explores the emerging possibility of using yeast directly as a probiotic organism. The human gastrointestinal tract (GIT) is an extreme environment for microbes due to its high acidity and competitive nutrition (Abolghasemi et al. 2022). The ability of several *Metschnikowia* species to endure harsh environmental conditions, such as salinity, high sugar content, and nitrogenous substrates, such as nectar, fermentation conditions, and insect guts, may contribute to the development of their survival-related strategies (Fig. 2) (Rodriguez Machado et al. 2024). These challenging conditions can stimulate the production of beneficial secondary metabolites, enabling them to monopolize nutrients; for instance, *M. reukaufii* in flower ecosystem (further elaboration in another chapter) (Dhami et al. 2016). Yeast survival mechanisms have likely contributed to the development of resilient species capable of thriving under extreme circumstances similar to those in the human intestine, as indicated by in vitro experiments (Abolghasemi et al. 2022; Smith et al. 2015). Initial evaluation of preselected *Metschnikowia* probiotic candidates focuses on their ability to persist in unfavorable GIT conditions, particularly in high-acidic situations, which serves as the first barrier in the GI tract. *Metschnikowia* exhibits probiotic characteristics in its native environment, including the regulation of other microorganisms’ growth under hostile conditions. Therefore, additional research on *Metschnikowia* as a probiotic is essential to refine its probiotic class and enhance its value in commercial applications. Current research on *Metschnikowia* has revealed general probiotic activities such as resistance to colonization in the GIT and competitive elimination of pathogens. Exploring more sophisticated activities like bile salt metabolism could be promising, considering that *Metschnikowia* species can tolerate bile (Abolghasemi et al. 2022; Rodriguez Machado et al. 2024; Smith et al. 2015; Staniszewski and Kordowska-Wiater 2023). In vivo studies are crucial to expedite the commercialization of *Metschnikowia* as a probiotic product. These studies would provide safety information to meet regulatory requirements for product manufacturing (Lahtinen et al. 2009).

## Promising secondary metabolites

Despite the diverse functional attributes revealed by scientists, understanding the metabolites produced by *Metschnikowia* yeast presents challenges and opportunities for exploration. Yeast cells produce secondary metabolites to protect themselves from pathogens or to adapt to unfavorable environmental conditions such as high/low temperatures, high salinity, and changes in sugar levels. Manipulating the growth medium or exposing *Metschnikowia* to other pathogenic organisms activates the production of protective metabolites. However, identifying natural metabolites in response to pathogens remains a challenge. Metabolomic techniques can be applied to better understand these complexities and to establish a high-quality comprehensive database of *Metschnikowia* metabolites. In this section, selected groups of secondary metabolites produced by *Metschnikowia* species are discussed to evaluate their potential application across agriculture, bioconversion, cosmetics, and pharmaceutical industries.

### Alkaloids

**Fig. 3:**
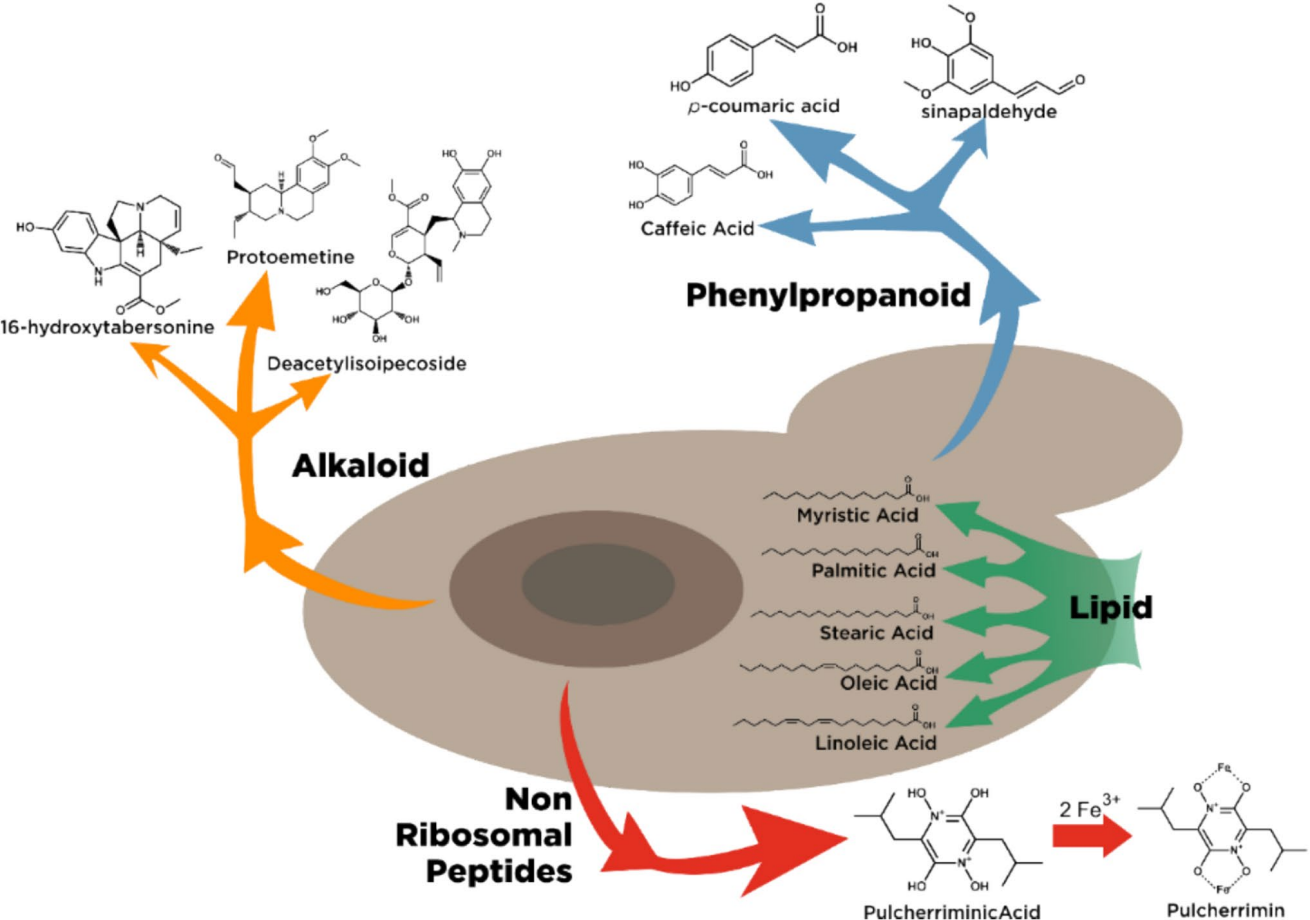
Selected promising secondary metabolites produced by *Metschnikowia* species

Although extensive research has been conducted on the exploration of secondary metabolites produced by *Metschnikowia* species, studies on the alkaloid production by this genus are lacking. However, the isolation of *Metschnikowia* species from toxic nectar comprising alkaloid compounds has been successful (Manson et al. 2007). *Metschnikowia gelsemii* (outdated name: *Candida galsemii*), a species isolated from toxic nectar, along with other species such as *M. pulcherrima* and *M. reukaufii*, was analyzed for gelsemine (an indole alkaloid) in yeast malt agar (Manson et al. 2007). Synthetic gelsemine (100 and 250 ng/µl) did not reduce the growth of the yeast (Manson et al. 2007). The presence of alkaloids in nectar may act as a defense mechanism against microbial organisms. For example, the alkaloids atropine and tropine, isolated from the nectar of *Atropa beetica*, had an inhibitory effect on yeast development (Pozo et al. 2012). Although gelsemine is a natural component of this nectar plant, the ability of *Metschnikowia* to grow in this unfavorable environment could be an attractive survival capability. The alkaloid tolerance of nectar by the genus *Metschnikowia* may be due to the processing mechanism of nitrogen compounds in the environment. For instance, *M. raukeufii* genetically facilitates the expression of amino acid-related genes (Dhami et al. 2016). These genes may be associated with alkaloid production, preventing the development of other organisms during the early stages. Production of *Metschnikowia* alkaloid in a liquid growth medium revealed the presences of deacetylisoipecoside, piperideine, protoemetine, and 16-hydroxytabersonine, identified using UPLC-MS (Fig. 3) (Millan et al. 2022). Four alkaloid compounds were produced by *M. pulcherrima* Mp-22 and Mp-30 in a mixture of yeast nitrogen base medium (YNBS) with glucose and ammonium sulfate and yeast extract-peptone dextrose medium. A possible explanation for this finding is that *Metschnikowia* possesses a gene cluster related to alkaloid biosynthesis, which acts as a survival mechanism against pathogens.

As indicated previously, both alkaloid tolerance and production are valuable starting points for the exploration of valuable alkaloid compounds in *Metschnikowia*. Understanding the mechanisms of action of the genus *Metschnikowia* under basic conditions could provide a new method to utilize this genus to produce alkaloid-active pharmacological compounds. However, studies on *Metschnikowia* have produced limited information till date about the alkaloid compounds natively produced by the yeast in nature. Further studies are needed to investigate the presence of not only native alkaloids but also enzymes that facilitate their biosynthesis. Approaching the biosynthetic pathway using bioengineered yeast, which produces plant alkaloids, may be a good starting point, even though alkaloids are a large group of secondary compounds with different precursors.

### Lipids

As previously mentioned, *Metschnikowia* showed an ability to accumulate lipid under adjusted culture conditions such as maintaining lower temperature and pH on limited nitrogen sources environment (Abeln and Chuck 2020; Tamilalagan et al. 2019). *Metschnikowia* can produce 30–40% (w/w) lipid of their dry mass (especially *M. pulcherrima*), which less than a typical oleaginous yeast, but it can endure high sugar content and enhanced lipid production in non-sterile environments (Fig. 3) (Abeln et al. 2020; Anbarasan et al. 2018; Santamauro et al. 2014). Those dual advantages are an attractive capability on production of biodiesel to substitute traditional fuel as sustainable and environmentally friendly energy resources (Leiva-Candia et al. 2014). *Metschnikowia* could incorporate numerous types of carbon sources, including distillery spent wash, food waste, animal fat, and volatile fatty acids (VFA), to generate the fatty acid methyl or ethyl ester, which is the major component of biodiesel (Abomohra et al. 2021; Anbarasan et al. 2018; Li et al. 2021; Nemcova et al. 2021). Moreover, under aerobic biotransformation process of organic waste substrate, *Metschnikowia* species could prevent the sterol uptake across plasma membrane through ABC transporters (Mbuyane et al. 2021). Excessive sterol deposit in yeast cell may have negative impact the quality on biodiesel during transesterification reaction such as filter blockage induced by sterol glycosides precipitation (Wang et al. 2010). The preliminary open-air cultivation of *M. pulcherrima* exhibited 34% of lipid, strengthening the capability of this yeast genus to culture under non-sterile circumstances with low agitation. (Santamauro et al. 2014). Moreover, *Metschnikowia* could produce a food-grade oil or lipids which is crucial in food manufacturing (Abeln et al. 2019). However, those findings also emphasized the significance of regulated culture conditions because growth and production potential are strain dependent (Abeln et al. 2019; Santamauro et al. 2014).

### Phenylpropanoid derivatives

Currently, reports on natural flavonoids or other phenylpropanoid derivatives produced by *Metschnikowia* species are unknown. Over the past few years, the most closely studied phenylpropanoid compound in this yeast has been used in enology and in biological control activity tests. In wine production, several yeasts have been co-cultured with *Metschnikowia* species as fermenters in the same substrate to investigate their effects on wine flavor and odor (Kelanne et al. 2020; Leonard et al. 2021; Liu et al. 2023; Puyo et al. 2023c). Grapes and berries are the most common substrates used in wine making; these fruits contain anthocyanins, flavonoids, and other phenolic compounds that are utilized by *Metschnikowia* species to increase phenolic levels (Fig. 3) (Kelanne et al. 2020; Liu et al. 2023; Puyo et al. 2023c). These findings support the hypothesis that *Metschnikowia* possess genes related to the phenylpropanoid biosynthesis pathway. Despite the presence of phenolic compounds as native ingredients in the substrate medium, *Metschnikowia* species successfully enhanced the concentration of these compounds during fermentation. *Metschnikowia pulcherrima* 70321 increased phenolic acid and flavonoid compounds in albino bilberry juice substrates (Puyo et al. 2023c). Furthermore, these yeast species increased the amount of caffeic acid and *p*-coumaric acid by approximately 25- and twofold, respectively. However, genes related to the phenylpropanoid biosynthetic pathway have not been identified in *Metschnikowia* till date. Transcriptomic methodology could reveal a biosynthetic pathway gene cluster, which could be activated as a defense response to unfavorable conditions such as the presence of pathogens. For instance, metabolite compound profiling of *M. pulcherrima* Mp-22 and Mp-30 culture filtrates revealed a cinnamaldehyde group named sinapaldehyde after dual culture with *Botrytis cinerea* in YNBS medium (Millan et al. 2022). This study could provide useful information for future studies exploring valuable native phenylpropanoid derivative compounds. Next-generation sequencing can be used to comprehensively study the phenylpropanoid biosynthetic pathway and its derivative compounds in the future.

### Pulcherriminic acid

*Metschnikowia* produces pulcherriminic acid, which is a non-ribosomal peptide with a cyclic dipeptide structure and shows a high affinity for ferric ions (Fe^3+^) to form red-brown pulcherrimin (Fig. 3) (Yuan et al. 2020). Pulcherriminic acid is not unique to *Metschnikowia* and is also produced by bacteria, such as *Bacillus subtillis* and *B. lichneiformis,* and yeasts. However, the enzymes responsible for pulcherrimin synthesis differ between bacteria and yeasts (Sipiczki 2020). *yvmC* and *cypX* contribute to the conversion of leucine tRNA into pulcherriminic acid, and *yvmA* facilitates the secretion of product compounds into the outer cell (Randazzo et al. 2016; Wang et al. 2018). The pulcherrimin (*PUL*) gene cluster, consisting of four genes, is responsible for pulcherrimic acid biosynthesis in yeast cells (Krause et al. 2018). The *PUL4* gene controls the expression of the *PUL1* and *PUL2* genes in the yeast *Kluyveromyces lactis* whereas the *PUL3* gene is responsible for the transport of the pulcherrimin by-product from the outer cell to the cell producer (Krause et al. 2018). The *PUL3* gene showed the same activity in* M. persimmonesis* in response to different growth media (Rahmat et al. 2024); it showed high expression in media with a high density of pulcherrimin, which represents a “metabolic signal” for switching the biosynthetic direction. In addition, culture media conditions have a strong effect on the expression of the *PUL* gene cluster, as evidenced by the increase in pulcherrimin formation following the addition of Tween-80 in the culture media for both *M. persimmonesis* and *M. pulcherrima* (Pawlikowska et al. 2020; Rahmat et al. 2024).

The production of pulcherriminic acid in *M. persimmonesis* is higher than that in *M. pulcherrima* in minimal medium supplemented with Tween-80 (Rahmat et al. 2024). The duplication of the genes *PUL1* and *PUL4* in the genome possibly promotes the production of pulcherriminic acid, which is also supported by Tween-80, resulting in improved transport of carbon into and out of yeast cells. Different species produce different amounts of secondary metabolites, which could be another area of research to optimize *Metschnikowia* species as a biological factory for pulcherrimic acid. Furthermore, enzymes from different yeast genera with high metabolism could be developed to produce pulcherriminic acid. The improvement of the pulcherriminic acid compound would contribute not only for biocontrol but also for its use as an antimicrobial agent against human pathogens. There is no scientific report which use pulcherriminic acid against human or plant pathogens. However, Kregiel et al. (2023) reported the pulcherrimin as single compound was able to suppress the growth of *Candida* and non-*Candida* (*Filobasidiella neoformans* var. *bacillispora* (syn. *Cryptococcus neoformans*)) species in the agar plate assay, while also exhibiting cytotoxicity and antiproliferative activity mediated by oxidative stress in the different cell line test. Moreover, antifungal capability of pulcherrimin will reduce in the presence of peptone and sodium silicate, indicated biological matrix affected their inhibition ability (Kregiel et al. 2023). Further investigation is required to establish the action mechanism of pulcherrimin in somatic cells, which contributes to the safety aspect.

## Metschnikowia-ecosystems interactions

The antimicrobial activity of *Metschnikowia* is related to its biological control of plant pathogens. However, biocontrol is a complex mechanism that involves plant, yeast, or bacterial pathogens, and interactions with infected plants. Biocontrol mechanisms of yeast against pathogens vary for each species and can involve nutrient competition between organisms, which is a simple and wide interaction between organisms that also affects other microorganisms. Moreover, *Metschnikowia* species show the capability to accumulate iron and nitrogen from the environment, which causes a nutrient deficiency in pathogenic microorganisms. The mechanisms underlying yeast biocontrol include increasing reactive oxygen species (ROS) activity, secreting lytic enzymes, forming a biofilm layer, and producing volatile compounds (Freimoser et al. 2019; Sipiczki 2020). The most extensively studied mechanisms of action are those of *M. citriensis* and *M. pulcherrima* (Wang et al. 2020; Yang et al. 2021), for which all in vitro biocontrol properties have been analyzed, in contrast to analysis of only one notable feature of other species.

**Fig. 4:**
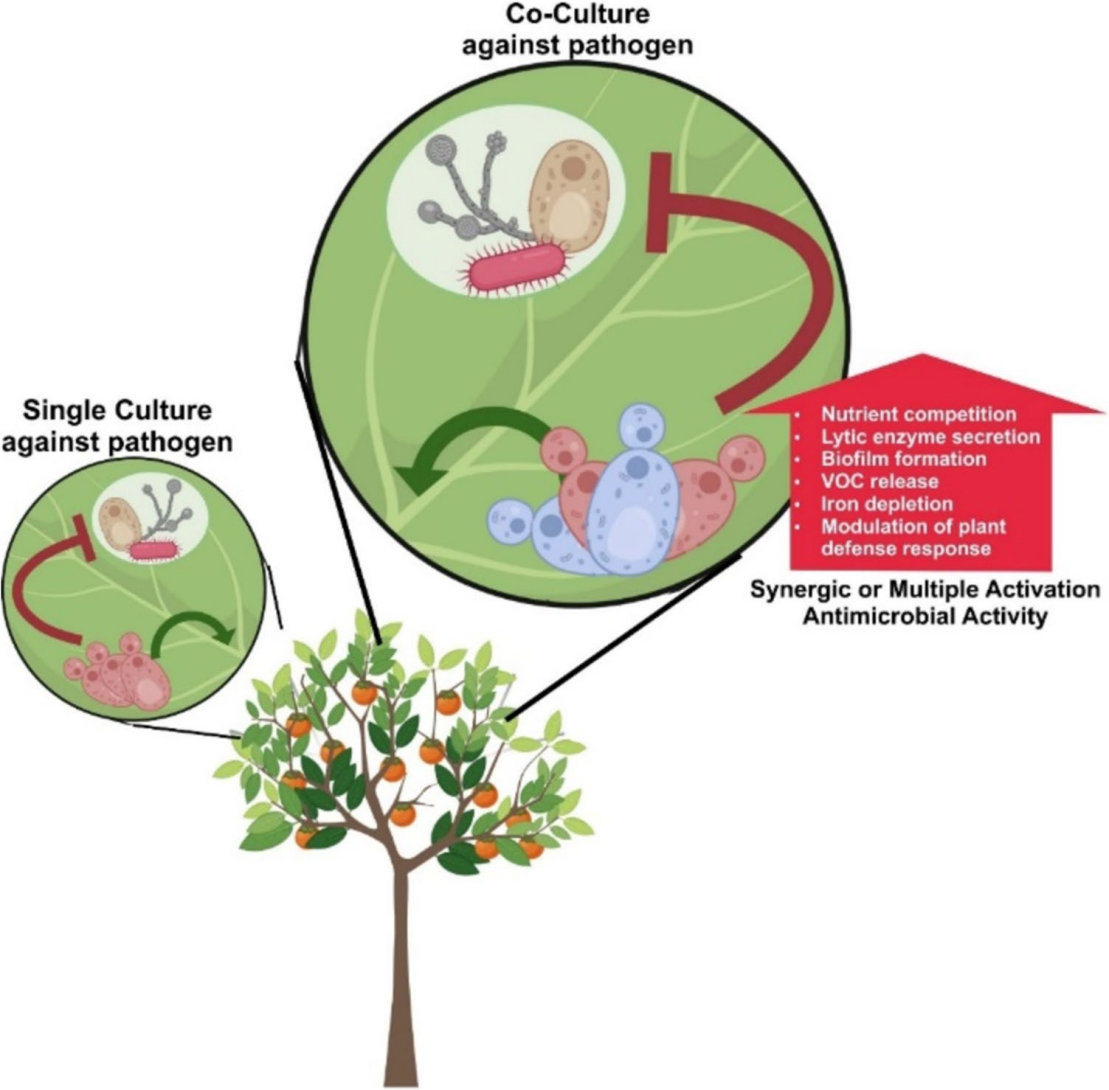
Schematic description of pathogen defense mechanisms of single or co-cultured *Metschnikowia*

*Metschnikowia* species are frequently co-isolated with other non-pathogenic yeasts that could contribute to antimicrobial activity (Öztekin and Karbancioglu-Guler 2023; Puyo et al. 2023a). These non-pathogenic yeasts, isolated from the same ecosystem as *Metschnikowia* species, could stimulate interaction with other organisms. For instance, *M. persimmonesis*, co-isolated with the common yeast *Hanseniaspora uvarum* (anamorph *Kloeckera apiculata*) in fruit ecosystems, presents an opportunity to study the interaction between the two different species in biological control activities (Choi et al. 2018). Although yeast-yeast interactions have been extensively studied in winemaking to enhance the quality of wine using non-pathogenic yeasts (Bordet et al. 2020; Comitini et al. 2021), similar investigations into yeast interactions as biocontrol agents are lacking (Fig. 4). A possible explanation for this gap may lie in the challenge of recreating natural growth environments for in vitro studies. Although traditional yeast media such as potato dextrose and lysogeny broth are suitable for yeast culture, other factors such as host-plant nutrition and unidentified organisms cohabiting in the same environment may inadvertently affect study outcomes. Therefore, a promising area of future research in biological control assay would be to investigate how *Metschnikowia* species interact with co-isolated yeasts in host plants or in other ecosystems against microbial pathogens. For instance, combination culture of *M. aff pulcherrima* PO1A016 with *Hanseniaspora uvarum* and *Meyerozyma guilliermondii* exhibited a synergistic biological control against *Penicllium digitatum* pathogen in mandarin fruit during in vitro study (Fig. 4) (Öztekin and Karbancioglu-Guler 2023).

Furthermore, *Metschnikowia* related to fruit ecosystem showed an interested interaction since biological control exhibited by *Metschnikowia* targeted fruit plant*. Metschnikowia* species isolated from fruit ecosystems account for almost 12% of those isolated from all ecosystems. The growth and survival of *Metschnikowia* on fresh fruits raises several questions: How do yeasts colonize the fruit? Where do they originate from? What substances in fruits attract *Metschnikowia* species to flourish in this ecosystem? Typically, the outer layer of fruits harbor a yeast population ranging from approximately 10^2^ to 10^6^ cfu/cm^2^ (Fleet 2003). Various external factors drive yeast colonization, including meteorological conditions that affect yeast adaptation to dynamic climate, application of agrochemicals, location of fruit cultivation, and fruit maturity (Fleet 2003). These factors affect not only the initiation of yeast colonization, but also the production of various secondary metabolites in response to exogenous dynamics. The fruit environment provides a variety of valuable nutrients that sustain basic metabolic activities for yeast survival. However, this environment offers limited amounts of nutrients, which likely induces the activation of secondary metabolite production as a protective mechanism. Fruits from which *Metschnikowia* species were first isolated include jujube (four species), grape (two species), peach (one species), and persimmon (one species). Recently, *Metschnikowia* species have been found on the surface of fruits, which are not their initial ecological systems, for example, *M. andauensis* has been found on the surface of apples, oranges, pomes, raspberries, strawberries, and grapes, possibly transferred by insects (Manso and Nunes 2011; Pawlikowska et al. 2019). The sweet taste of fruits, owing to sugar production on their outer surfaces, renders them ideal environments for *Metschnikowia* growth. Yeast cells secrete extracellular enzymes, such as glycosidases, which are responsible for sugar metabolism, and proteases, which break down amino acids in the outer layer of fruits, leading to the production of various secondary metabolites, such as alkaloids or phenolic acids (Kelanne et al. 2020; Millan et al. 2022). Moreover, the application of synthetic chemical pesticides to fruits might alter the synthesis of secondary metabolites in the plants, and have a potentially similar effect on yeast metabolism. Several scientific reports have been revealed that application of synthetic pesticide on both aerobic and anaerobic condition significantly decreased the growth rate in yeast, potentially inducing the production of distinct secondary substances as a protective response (Becerra et al. 2023; Jawich et al. 2005, 2006). Pesticide treatment at the maximum residue level (MRL) significantly influences several metabolic pathways in yeast, including those of alanine, arginine, aspartate, and glutamate (Song et al. 2022). Amino acid-related metabolic pathways affected by xenobiotics are generally assumed to play a role in the production of protective compounds during yeast metabolism, such as true and proto-alkaloid types, which are nitrogen derived from amino acid compounds, might affect yeast transcription dynamics in the presence of pesticides. Moreover, combination study of *Metschnikowia* with both antagonistic organism and organic biological treatments exhibited significantly reduction of both the incidence of grey mold and powdery mildew under open field vineyard conditions (Agarbati et al. 2022; Lombardo et al. 2023). The synergism interaction between *Metschnikowia* with non-chemical pesticide possibly altered various mechanism which strengthen the antimicrobial activity on the specific environment (Fig. 4).

The terrestrial ecosystem was the most initial habitat of *Metschnikowia* yeast to be discovered, and included plant parts such as leaves, flowers, fruits, and bark, highlighting the relationship between ecosystem and yeast utilization (Table S1**, **Fig. 1). For instance, *M. koreensis* and *M. persimmonesis*, native to the Korean Peninsula, represent initial biogeographic discoveries, and are found in a variety of terrestrial environments corresponding to distinct ecosystems. *Metschnikowia koreensis* was initially discovered in the flower sections of *Lilium* sp. and *Ipomea* sp., whereas *M. persimmonesis* was isolated from the calyx of persimmon fruits. Notably, these species benefit humans in different ways. Research on the biotransformation activity of *M. koreensis* has generated interest in prospective applications because of the capability of this species to accumulate in flower and nectar environments, which are extreme environments for microorganisms due to the lack of essential nutrients. *Metschnikowia koreensis* possesses a reducing enzyme that plays an important role in bioconversion (Meena et al. 2014; Sasahara and Izumori 2005; Singh et al. 2011). In contrast, *M. persimmonesis* synthesizes pulcherriminic acid, which forms complex ferrous materials in the environment. Ferrous ion competition leads to nutrient deprivation, resulting in inhibition of pathogen growth. These organisms naturally produce a variety of chemicals in response to external signals. In response to the presence of pathogens, *M. persimmonesis* secretes benzene acetic acid, benzoic acid, 4-hydroxyl-benzoic acid, 4-hydroxyl-benzaldehyde, lumichrome, 4-(2-hydroxyethyl) benzoic acid, cyclo(Leu-Leu), heptadecanoic acid, and non-adecanoic acid which probably valuable entities as biopesticide in the future. In contrast, as a biotransformation species, *M. koreensis* converts several precursors to valuable compounds through a stereoselective reduction process or a general redox reaction, producing compounds such as secondary alcoholic diol and L-psicose. This transformation capability arises in response to the presence of non-indigenous compounds. *M. koreensis* has been effectively isolated in other nations, including Mexico and Thailand (Canto et al. 2021; Limtong and Kaewwichian 2015). These findings support the theory that *M. koreensis* was initially discovered and recognized in Korea, although it is not the only indigenous species of *Metschnikowia* found there. Moreover, *Metschnikowia*'s ecosystem of origin may provide information to the species' implementation in daily life, as every habitat fosters unique survival strategies that can be exploited.

## Current and future commercialization of Metschnikowia

The massive production of yeast, as the main ingredient of commercial end products or as biological factories to produce several valuable compounds, faces a significant obstacle. Stringent regulatory requirements aimed at ensuring consumer safety pose a challenging hurdle in industrial production. This aspect is understandable for industrial companies because the capability to manufacture a safe product can increase its value. Nevertheless, owing to its non-pathogenic quality, *Metschnikowia* is widely used as a starter culture in the winemaking industry, as a biological control in the horticulture field to prevent molding caused by plant-specific pathogens, and as an active ingredient in the cosmetic industry (EFSA et al. 2017; Jolly et al. 2014; Paufique 2013, 2019; Roudil et al. 2020; Vicente et al. 2020). In this section, we discuss the current applications of *Metschnikowia* species, along with the regulatory constraints and challenges they face. In addition, the prospective field of the industrialization of *Metschnikowia* and its challenges are discussed.

*Metschnikowia pulcherrima* and *M. fructicola* serve as commercial starting fermenters for wine production, each contributing differently to wine production, highlighted two advantages as bioprotectant or aroma enhancer (Table 1) (Binati et al. 2023; Canonico et al. 2023; Escott et al. 2022; Bastien 2021; Naselli et al. 2023; Puyo et al. 2023b; Varela et al. 2021). The application of *Metschnikowia* to control native microorganisms or improve aroma compounds needs to fulfill the safety requirements of the International Organization of Vine and Wine. Monographs of *Saccharomyces* and non-*Saccharomyces* yeasts have been included in the International Oenological Codex since 2017, describing an important role for both yeasts in the winemaking process (OIV 2024). The commercial product is required to meet the limits for moisture content, heavy metals, and microbial contamination, as stated in the codex. Briefly, the requirements for non-*Saccharomyces* yeasts for wine production are related to safety, such as heavy metal and microbial contamination. In addition, the total viable yeast and the identification of the contaminating taxa is essential to maintain the quality of the product from batch to batch. This codex requires the contaminant population to be < 5% of the total population, and stipulates determination of the genus and species/strains of the contaminant. These conditions focus more on the safety of the product instead of quality. However, both the safety and quality of commercial products consumed by humans are required.

As shown in Fig. 2, most *Metschnikowia* species exhibit biological control activity. However, only one commercial product, traded under brand name Noli® and Shemer®, uses *M. fructicola* as a biocontrol agent in crop fields (Table 1). This product can be applied to grapes and soft fruits, such as strawberries and blueberries, to effectively control *Botrytis* incidence rates. In addition, it can be used to prevent the severity of *Monilla* brown rot disease in stone fruits, such as cherries and plums, as claimed by the company. As a biological control entity, the impact of biopesticide usage on the environment, especially on biotic organisms, might be the most critical point to consider before a distribution license is granted. Hence, the safety aspect of the long-term usage of biocontrol agents should be an additional requirement for inclusion in the current regulations. The successful application of biological pesticides is dependent on living organisms in the ecosystem, which might cause a hindrance to the application. The regulatory institution authorized to permit the commercialization of biocontrol should consider the instability of population organisms affected by biopesticides. Post-market surveillance regulations for pharmaceutical items can be adopted by policy makers to monitor the long-term impacts of biopesticides on the environment (particularly microbial balance) and human health. Moreover, the acute and chronic toxicities of the application biopesticide should considered by policy maker during registration process.

According to the above discussion, regulation poses a critical obstacle to the application of *Metschnikowia*, or microorganisms in general, as commercial products. Despite increasing exploration of other biological substances, especially microorganisms, as alternatives to chemical pesticides, clear regulations have not been uniformly established across all types of commercial products (Table 2). Compiling appropriate regulations for each commercial product is a complicated process because of the different interests of each stakeholder, despite the broad consensus on product safety. In addition to the food, cosmetic, and pharmaceutical products based on *Metschnikowia*, it is almost certain that more commercial products are produced by non-pathogenic microorganisms (Table 2). Therefore, regulations in this field can be adapted from existing rules for other applications that require efficacy activity tests as a criterion. For instance, microorganisms frequently used as probiotics to maintain the microflora in the gastrointestinal tract could be considered a model system. Notably, *Metschnikowia* species show antimicrobial activity against human pathogens; therefore, further exploration of the human applications of *Metschnikowia* is warranted. For regulatory purposes, the safety, efficacy, and quality of *Metschnikowia* in human applications can follow those of similar products, having the same ingredients and efficacy category.

**Table 2:** List of *Metschnikowia* yeast species with promising into commercial product

*Metschnikowia* species	Industrial field	Activity	References
*Metschnikowia citriensis*	Agriculture	Antifungal	Zeng et al. (2020)
*Metschnikowia persimmonesis*	Agriculture	Antifungal	Rahmat et al. (2024)
*Metschnikowia rubicola*	Cosmetics	Unclear	Franchi et al. (2023)

## Conclusion

More than a century has passed since yeast of the genus *Metschnikowia* was first discovered. Considerable progress has been made in the sequential identification of new species belonging to this genus and exploring their potential usefulness for human life. *Metschnikowia* strains are found in both aquatic and terrestrial environments, with most aquatic species being parasitic on other living organisms. In contrast, *Metschnikowia* species found in isolated land areas exhibit beneficial characteristics for both human life and the environment. These *Metschnikowia* species have functional properties that can be used not only for biological control against pathogens but also for various other purposes. Currently, various products derived from *Metschnikowia* strains are being developed and commercially distributed for limited applications, including agriculture, food production, cosmetics, and pharmaceutical manufacturing. As these products contain microorganism-derived materials, it is necessary to accurately and extensively review the safety and potential risks of these final products to ensure continued safe usage in the future. Furthermore, establishing a rational regulatory framework for the utilization of these products is essential to ensure user safety and environmental preservation.

## Supplementary Information

Below is the link to the electronic supplementary material.

Supplementary file1 (XLSX 19 KB)

## Data Availability

This article’s initial data can be accessed from https://www.mycobank.org and https://www.indexfungorum.org.
